# ToPP: Tumor online prognostic analysis platform for prognostic feature selection and clinical patient subgroup selection

**DOI:** 10.1016/j.isci.2022.104190

**Published:** 2022-04-04

**Authors:** Jian Ouyang, Guangrong Qin, Zhenhao Liu, Xingxing Jian, Tieliu Shi, Lu Xie

**Affiliations:** 1 The Center for Bioinformatics and Computational Biology, Shanghai Key Laboratory of Regulatory Biology, the Institute of Biomedical Sciences and School of Life Sciences, East China Normal University, Shanghai 200241, China; 2 Shanghai-MOST Key Laboratory of Health and Disease Genomics, Institute for Genome and Bioinformatics, Shanghai Institute for Biomedical and Pharmaceutical Technologies, Shanghai 200237, China; 3 Institute for Systems Biology, Seattle, WA 98109, USA; 4 Bioinformatics Center, National Clinical Research Centre for Geriatric Disorders, Department of Geriatrics, Xiangya Hospital, Central South University, Changsha, Hunan 410083, China; 5 Big Data and Engineering Research Center, Beijing Children’s Hospital, Capital Medical University, National Center for Children’s Health, Beijing 100045, China

**Keywords:** Oncology, Public health, Microbiome

## Abstract

Patients with cancer with different molecular characterization and subtypes result in different response to anticancer therapeutics and survival. To identify features that are associated with prognosis is essential to precision medicine by providing clues for target identification, drug discovery. Here, we developed a tumor online prognostic analysis platform (ToPP) which integrated eight multi-omics features and clinical data from 68 cancer projects. It provides multiple approaches for customized prognostic studies, including 1) Prognostic analysis based on multi-omics features and clinical characteristics; 2) Automatic construction of prognostic model; 3) Pancancer prognostic analysis in multi-omics data; 4) Explore the impact of different levels of feature combinations on patient prognosis; 5) More sophisticated prognostic analysis according to regulatory network. ToPP provides a comprehensive source and easy-to-use interface for tumor prognosis research, with one-stop service of multi-omics, subtyping, and online prognostic modeling. The web server is freely available at http://www.biostatistics.online/topp/index.php.

## Highlights

• ToPP platform integrated eight multi-omics and clinical data from 68 cancer projects
• ToPP provides multi-omics combination and subgroup selection for prognostic analysis
• ToPP provides automatic construction of prognostic model for public and custom data
• Users can perform prognostic analysis based on regulatory network or pathways in ToPP

## Graphical abstract

**Figure:**
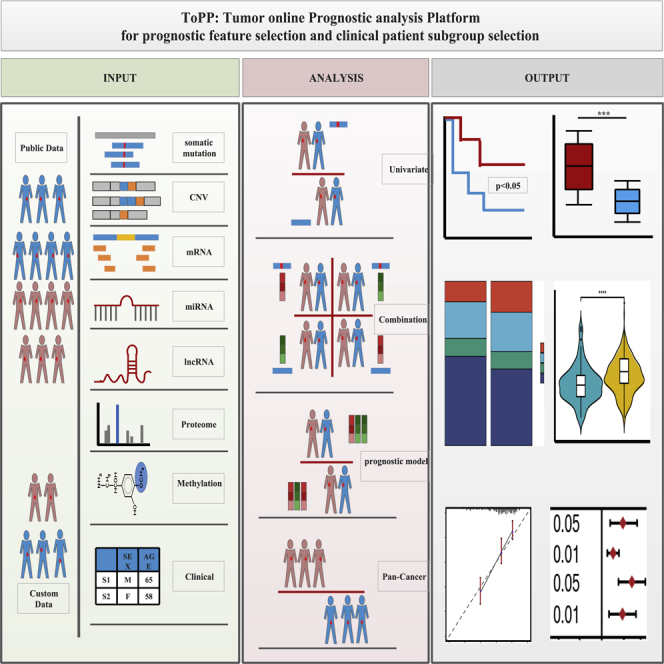

## Introduction

Cancer is among the top fatality diseases which cause approximately 10.0 million deaths worldwide each year (Sung et al., 2021). Prognosis analysis is the prediction of the probable outcome from an individual’s current medical condition (Hansebout et al., 2009). Many factors are closely related with prognosis in cancer, including age, tumor types, pathological stages, genetic background, and molecular features at different levels (Banfalvi, 2014; Montazeri, 2009). Prognosis analysis from large patient cohort can help identify biomarkers or potential targets for patients with different clinical outcomes, and it can also evaluate the outcomes of patients with different treatments or in different subgroups. However, it is cumbersome for clinical researchers to conduct prognosis analysis due to lack of follow-up data, clinical data, or statistical skills.

Recently, with the efforts from large consortia, such as The Cancer Genome Atlas (TCGA) (Weinstein et al., 2013), International Cancer Genome Consortium (ICGC) (J. Zhang et al., 2019), and the Clinical Proteomic Tumor Analysis Consortium (CPTAC) (Zhang et al., 2016b), tremendous amount of clinical and omics data have been available to the research community, while the analysis and utilization of these data are still difficult due to the large data size. Several prognosis analysis platforms have been developed. PrognoScan (Mizuno et al., 2009) focuses on the microarray datasets of 14 tumors, and provides the minimum log-rank p-value by the best cut-off point. GEPIA (Tang et al., 2017) provides not only patient survival analysis but also differential expression analysis, correlation analysis, and dimensionality reduction analysis based on gene expression. Oncolnc (Anaya, 2016) contains the relationship between lncRNA, miRNA, mRNA, and prognosis in 21 types of tumors. CaPSSA (Jang et al., 2019) provides prognostic analysis at the gene expression and mutation levels. MethSurv (Modhukur et al., 2018) is a web server for survival analysis based on methylation data in 25 tumor types in TCGA. However, most of them focused on the prognostic impact of genetic changes at a single level or provide prognosis with fixed subgroups. Practically, it is not robust to evaluate the prognosis of all patients through single prognostic marker due to the complexity of the disease and the heterogeneity of the patients, so it is necessary to establish a more customized, comprehensive, and effective prognostic analysis platform with joint analysis of multi-omics data for user-defined subgroups.

To bridge the gap between the high demand for versatile prognosis analyses from clinical researchers and the big multi-omics datasets, here, we developed the tumor online prognostic analysis platform (ToPP), which provides prognostic analysis using multi-omics (genome, transcriptome, proteome, and epigenome) data and clinical data in both the univariate and multivariate modules. Furthermore, users can build prognostic models or perform pan-cancer prognostic analysis on this platform by submitting their own data. For promoting the application in precision medicine, we also provide in-depth investigation of regulatory relationship or molecular mechanism among prognostic features, as illustrated by four sophisticated case studies on a more precise tumor subgroup stratification.

## Result

### Overview of ToPP

**Figure 1:**
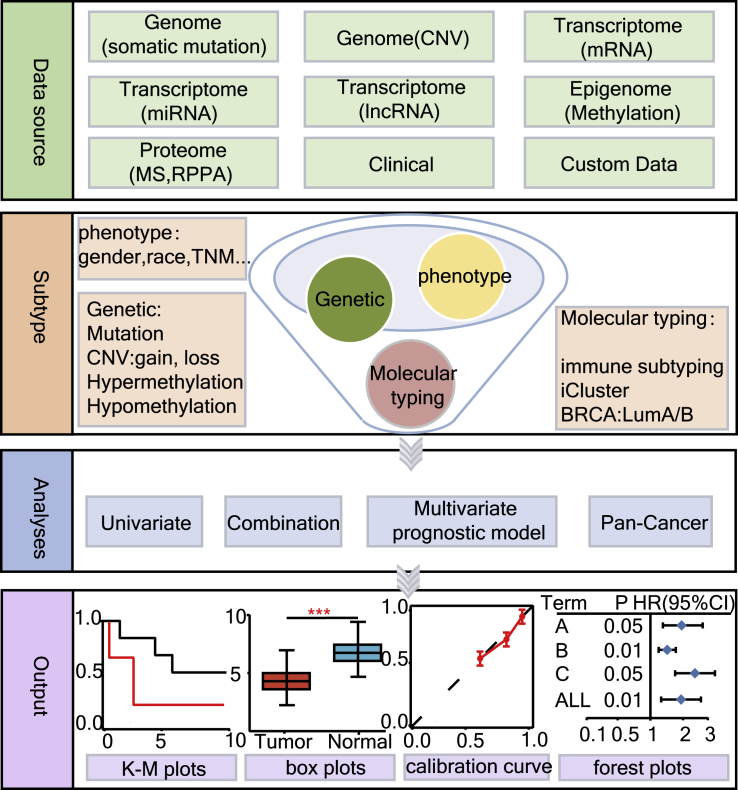
The overview of ToPP workflow The data source part shows the datatype collected in ToPP such as genome, transcriptome, proteome, epigenome, and clinical data; the subtype part shows the way for subgroup selection; the analyses part shows the function module in ToPP; and the output part shows the schematic diagram for the results.

The overview of ToPP is illustrated in Figure 1. ToPP usage mainly includes four steps: data aggregation, patient stratification, prognosis modeling, and result visualization. ToPP aggregates eight types of multi-omics data and clinical data (Table 1) from 33 tumors in TCGA (Table 2), CPTAC and 35 projects in ICGC. It also allows users to upload their in-house data for prognosis analysis. ToPP provides patient stratification through multiple ways. Users can select three conditions when conducting subgroup selection. The phenotype conditions include the patient's clinical data, mainly in physiological and experimental indicators. The molecular conditions are indicators related to molecular characteristics that have been widely used in various tumors, including specific molecular subtypes, immunophenotyping, etc. The genetic conditions are customizable; users can screen specific patient subgroups through a certain molecular feature of interest, thus achieving precise subtyping. Four prognosis modules were provided in ToPP, namely univariate module, multivariate module, pan-cancer module, and combination module, which will be illustrated in the following sections. ToPP also provides a variety of customized charts, such as KM curve, box plot, and calibration curve.

**Table 1:** Data sources collected in ToPP

	data type	Source	URL
Genome	Mutaion	UCSC Xena	http://xena.ucsc.edu/
		ICGC data portal	https://icgcportal.genomics.cn/
	CNV	cBioPortal	https://www.cbioportal.org/
		ICGC data portal	https://icgcportal.genomics.cn/
	Fusion	TumorFusions	http://www.tumorfusions.org
Transcriptome	mRNA	UCSC Xena	http://xena.ucsc.edu/
		ICGC data portal	https://icgcportal.genomics.cn/
	miRNA	UCSC Xena	http://xena.ucsc.edu/
		ICGC data portal	https://icgcportal.genomics.cn/
	LncRNA	TANRIC	https://ibl.mdanderson.org/tanric/_design/basic/index.html
Proteome	RPPA	UCSC Xena	http://xena.ucsc.edu/
	iTRAQ	CPTAC	https://cptac-data-portal.georgetown.edu/cptacPublic/
Epigenome	Methylation	UCSC Xena	http://xena.ucsc.edu/
		ICGC data portal	https://icgcportal.genomics.cn/
Clinical data	Phenotype &Follow up	UCSC Xena	http://xena.ucsc.edu/
		cBioPortal	https://www.cbioportal.org/
		ICGC data portal	https://icgcportal.genomics.cn/

**Table 2:** Multi-omics data of 33 types of cancer from TCGA included in ToPP

Type	Genome		Transcriptome				Proteome		Epigenome	Clinical data
	Mutation	CNV	Fusion	mRNA	miRNA	LncRNA	RPPA	MS	Methylation	Phenotype
TCGA-ACC	√	√	√	√	√	NA	√	NA	√	√
TCGA-BLCA	√	√	√	√	√	√	√	NA	√	√
TCGA-BRCA	√	√	√	√	√	√	√	√	√	√
TCGA-CESC	√	√	√	√	√	√	√	NA	√	√
TCGA-CHOL	√	√	√	√	√	NA	√	NA	√	√
TCGA-COAD	√	√	√	√	√	√	√	√	√	√
TCGA-DLBC	√	√	√	√	√	NA	√	NA	√	√
TCGA-ESCA	√	√	√	√	√	NA	√	NA	√	√
TCGA-GBM	√	√	√	√	√	√	√	NA	√	√
TCGA-HNSC	√	√	√	√	√	√	√	NA	√	√
TCGA-KICH	√	√	√	√	√	√	√	NA	√	√
TCGA-KIRC	√	√	√	√	√	√	√	NA	√	√
TCGA-KIRP	√	√	√	√	√	√	√	NA	√	√
TCGA-LAML	√	√	√	√	√	NA	NA	NA	√	√
TCGA-LGG	√	√	√	√	√	√	√	NA	√	√
TCGA-LIHC	√	√	√	√	√	√	√	NA	√	√
TCGA-LUAD	√	√	√	√	√	√	√	NA	√	√
TCGA-LUSC	√	√	√	√	√	√	√	NA	√	√
TCGA-MESO	√	√	√	√	√	NA	√	NA	√	√
TCGA-OV	√	√	√	√	√	√	√	√	√	√
TCGA-PAAD	√	√	√	√	√	NA	√	NA	√	√
TCGA-PCPG	√	√	√	√	√	NA	√	NA	√	√
TCGA-PRAD	√	√	√	√	√	√	√	NA	√	√
TCGA-READ	√	√	√	√	√	√	√	√	√	√
TCGA-SARC	√	√	√	√	√	NA	√	NA	√	√
TCGA-SKCM	√	√	√	√	√	√	√	NA	√	√
TCGA-STAD	√	√	√	√	√	√	√	NA	√	√
TCGA-TGCT	√	√	√	√	√	NA	√	NA	√	√
TCGA-THCA	√	√	√	√	√	√	√	NA	√	√
TCGA-THYM	√	√	√	√	√	NA	√	NA	√	√
TCGA-UCEC	√	√	√	√	√	√	√	NA	√	√
TCGA-UCS	√	√	√	√	√	NA	√	NA	√	√
TCGA-UVM	√	√	√	√	√	NA	√	NA	√	√

∗ RPPA: Reverse phase protein array; √: with data; NA: without data.

### Prognosis modules

The univariate module focuses on the impact of a single variable (genetic or clinical) on prognosis. Genetic features contain gene or protein expression changes, DNA variation, and epigenome changes. Users can select the datasets, data types, and variables to their interest. If the variable is continuous, the default patient stratification strategy is to use the median value for this variable (eg. Gene expression value) across all samples selected as a cutoff. In clinical research, especially in the demand of precision medicine, researchers may focus on the performance of prognostic factors in a particular subgroup, we therefore developed conditional screening tool to meet the subgroup screening. The conditional screening is based on gene mutation, methylation changes, or clinical classification. The univariate module provides a KM curve, the log-rank p-value, and hazard ratio of the two groups of patients. Meanwhile, users can make adjustments to the KM curve visualization, such as risk table, group color, unit time, median survival, and hazard ratio (HR). Furthermore, boxplot for the gene expression in tumor and normal groups can be drawn if the user sets the radio named differential expression to “yes”.

Multivariate module provides prognosis analysis for multiple variables, such as for a given gene list. The result for multivariate module contains a table with five columns: gene list, univariate analysis log-rank p-value and hazard ratio, and multivariate analysis log-rank p-value and hazard ratio for each gene, so that users can screen out the independent prognostic factors. In addition, it also provides the function of prognostic modeling, which includes model building, model evaluation, model diagnosis, and model validation. With the input of a gene list, the selection of training set and verification set, ToPP will automatically build the model by default parameters which are set by common processes. After checking the performance of the model in the training set and validation set (such as the three- or five-year calibration curve, C-index and 1000 bootstrap validator) and the results of the model diagnosis (PH assumption, outliers, and nonlinearity), users can adjust the model accordingly by further variable selection, nonlinear transformation. Users can also consider the interaction of covariates or time-dependent covariates.

Pan-cancer module was designed to investigate the prognostic effects of factors in a variety of tumors. All data were acquired from the Pan-Cancer Atlas (Hoadley et al., 2018) with unified normalization and standardization. Users can select all types of cancer or just choose a subset (Table S2) of pan-cancers such as urologic (bladder urothelial carcinoma [BLCA], prostate adenocarcinoma [PRAD], testicular germ cell tumors [TGCT], kidney renal clear cell carcinoma [KIRC], kidney chromophobe [KICH], and kidney renal papillary cell carcinoma [KIRP]) which was designed by Pan-Cancer Atlas project. The following up patient stratification and prognosis modeling approach are as the same as the univariate module. Besides, ToPP also includes the high frequently mutated gene lists in the ten canonical pathways which are, cell cycle, Hippo, Myc, Notch, Nrf2, PI-3-Kinase/Akt, RTK-RAS, TGFβ signaling, p53, and beta-catenin/Wnt (Sanchez-Vega et al., 2018), and it can facilitate users to explore how the alterations (copy-number alterations, mutations, fusions, or epigenetic silencing) of genes in these pathways impact prognosis in different tumor types. The results will show the log-rank p, HR, and KM curve for each type of cancer as well as the pan-cancer.

Combination module allows users to input multiple levels of data for two genes separately. It allows for more precise stratification of patients. For example, given a condition of mutation of one gene, researchers may want to know whether the mutation or expression of its regulated gene will have an impact on the patient's survival. Through the combination module, the samples would be subdivided into four subgroups; each subgroup has more clear molecular characteristics. By analyzing whether there are significant differences in survival among the four subgroups, users can carry out molecular subtyping of patients.

#### Data upload module

Data upload module allows users to upload their own data with survival time and status to conduct all kinds of analysis in ToPP. Also, users are advised to set the permission limit for their own data and use an e-mail for contact if necessary.

### Case studies in ToPP

#### Lipid metabolism affects cancer survival

**Figure 2:**
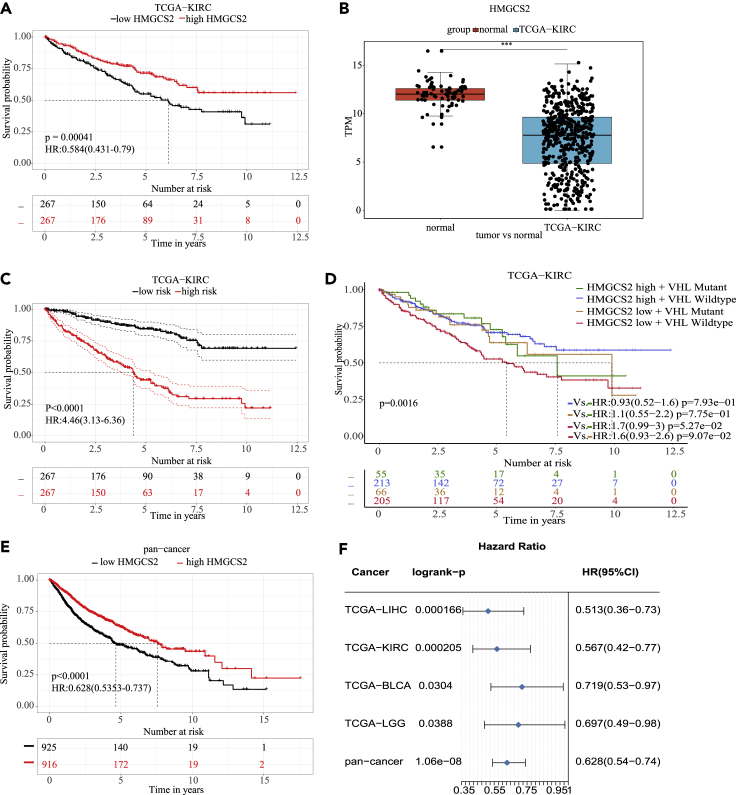
Research on lipid-metabolism-related genes in ToPP (A) The univariate analysis module results show that patients with kidney renal clear cell carcinoma (KIRC) with lower expression of HMGCS2 have poor prognostic (log-rank test, p = 0.00041). (B) KIRC samples have lower expression compared with normal samples (p < 0.0001, wilcoxon rank sum test). (C) Multivariate analysis module results show that the genes which are significantly associated with prognosis of patients with tumor in PPAR pathway (CPT2, ACADL, FADS2, CPT1B, CPT1C, ACOX3, CYP27A1, LDLR, ANGPTL4, CD36, SLC27A2, ACOX2, FABP6, HMGCS2, PLIN4, PLIN5, PPARG, MMP1, PCK1, PLIN2, GK, NR1H3, PPARD, OLR1, RXRB, PDPK1, and RXRA) have a significant impact on the prognosis of patients with KIRC (log-rank test, p < 0.0001). (D) The Combination module reveals that the KIRC subgroup of patients with low expression of HMGCS2 and no somatic mutation in VHL may have the highest risk (log-rank test, p = 0.0016). (E and F) HMGCS2 has the similar pattern in a variety of tumors including liver hepatocellular carcinoma (LIHC), bladder urothelial carcinoma (BLCA), brain lower-grade glioma (LGG), and KIRC (log-rank test, p < 0.0001).

Our previous study has shown lipid metabolism in cancer shows significant prognosis effects (Hao et al., 2019). PPAR signaling pathway is a key signaling pathway that regulates lipid metabolism. Both peroxisome proliferator-activated receptor (PPAR) α and PPARγ were reported to regulate the expression of 3-hydroxy-3-methylglutaryl CoA synthase 2 (HMGCS2) (Kim et al., 2019; VilÃ -Brau et al., 2011). To comprehensively investigate the impact of PPAR signaling pathway and HMGCS2 on patient outcomes, we performed analyses using multiple modules in ToPP. The univariate analysis result show that patients with kidney renal clear cell carcinoma (KIRC) with lower expression of HMGCS2 are associated with worse prognosis (Figure 2A) and it shows downregulation in tumor samples compared with normal samples (Figure 2B). In order to study the influence of the gene sets on the prognosis, the gene list in PPAR signaling pathway which related to tumor prognostics as we previous reported (Hao et al., 2019) were inputted for multivariate analysis in KIRC dataset, and our results show the different status of PPAR signaling pathway can distinguish the prognosis of patients with KIRC (Figure 2C). To address the combinatory prognostic effects for HMGCS2 and other driver genes, we also make a combinatory prognostic analysis using the combinatory module. Von Hippel-Lindau (VHL) is the most frequently mutated gene in patients with KIRC; we then performed the combination analysis for the VHL and HMGCS2. Interestingly, patients who have lower expression of HMGCS2 and with no mutation in VHL show poorer prognosis than other subgroups (Figure 2D). In order to explain this result, we propose two potential hypotheses through literature research. First one is that VHL directly interacted with and promoted ubiquitination of PPARγ leading to its degradation, and inhibition of PPARγ reduced HMGCS2 expression (Hernandez-Quiles et al., 2021; Kim et al., 2019; Noh et al., 2020). The other is that VHL is suggested as mutated as an early event for tumor, and HMGCS2 may have a negative regulation of tumor angiogenesis (Gossage et al., 2015; Zou et al., 2019). And their impact on prognosis may be independent. For more evidence to confirm these hypotheses, researchers need to do more experiments to verify them. To further investigate whether HMGCS2 is associated with prognosis in different tumor types, we perfomed the pan-cancer analysis for HMGCS2 in ToPP using the pan-cancer analysis module. The results show that HMGCS2 has the similar pattern in a variety of tumors (Figures 2E and 2F), including liver hepatocellular carcinoma (LIHC), bladder urothelial carcinoma (BLCA), brain lower-grade glioma (LGG), and KIRC.

#### Regulatory relationship investigation through multi-omics prognostic analysis in ToPP

**Figure 3:**
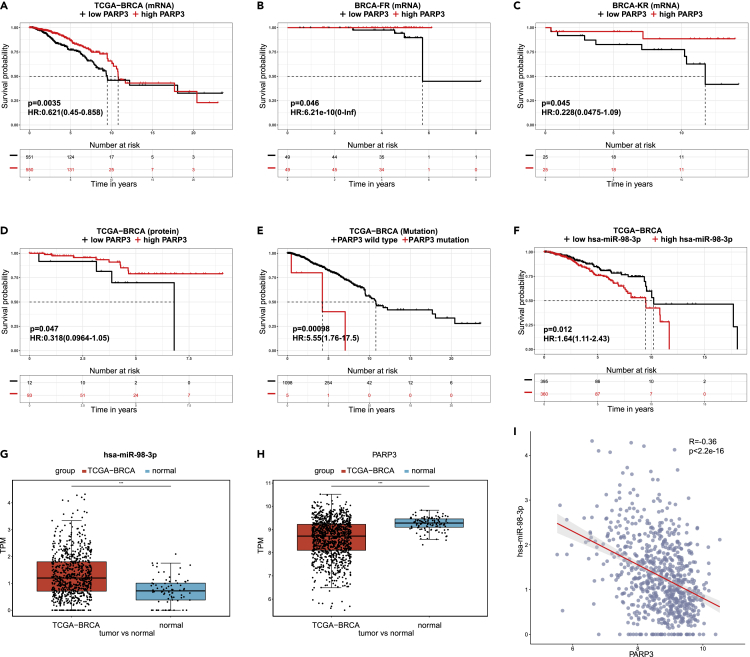
Multi-omics prognostic analysis in ToPP (A) Prognosis analysis of PARP3 mRNA expression in TCGA-BRCA cohort, patients with higher expression of PARP3 have better prognosis (log-rank test, p < 0.0035, HR: 0.621 95%CI: 0.45–0.858). So it is in two independent datasets (B and C). (D) The prognosis analysis of expression of PARP3 in protein level (iTRAQ) also indicates PARP3 is a protection factor to patients with breast cancer (log-rank test, p = 0.047). (E) Patients with PARP3 mutation may have poor prognosis (log-rank test, p = 0.00098). (F) High expression of hsa-miR-98-3p was significantly associated with poor prognosis in patients with breast cancer (log-rank test, p = 0.012). (G) The expression for hsa-miR-98-3p in BRCA tumor tissue and adjacent normal tissue (wilcoxon rank sum test, ∗∗∗, p < 0.001). (H) The expression for PARP3 in BRCA tumor tissue and adjacent normal tissue (wilcoxon rank sum test, ∗∗∗, p < 0.001). (I) Correlation between the expression of hsa-miR-98-3p and PARP3 in tumor tissue (spearman correlation, p < 2.2e-16, R = −0.36).

Poly (ADP-ribose) polymerase 3 (PARP3) is the third member of the PARP family and it can accelerate the repair of chromosomal DNA single-strand breaks (Grundy et al., 2016). Researches reported that PARP3 is a driver gene to TGFβ-induced epithelial-to-mesenchymal transition and its inhibitors sensitize breast cancer cells to vinorelbine which was used in the treatment of metastatic breast cancer (Beck et al., 2019; Sharif-Askari et al., 2018). Here, we investigate the prognosis effect of PARP3 in patients with breast cancer. Firstly, we explored the relationship between PARP3 expression and overall survival in ToPP, and the result showed that patients with higher expression of PARP3 had a better prognosis than the patients with lower expression of PARP3 in TCGA-BRCA cohort (p = 0.0035, Figure 3A). This conclusion could be verified in other two independent datasets BRCA-FR and BRCA-KR in ICGC Data Portal (p = 0.046, Figure 3B, p = 0.045, Figure 3C). Then, we further investigated the prognosis effect of PARP3 in patients with breast cancer with both proteomic and genomic data, and the results were consistent, which mean that PARP3 is still a protection factor to patients in proteomic level (p = 0.047, Figure 3D). Meanwhile, patients with PARP3 mutation have a poor prognosis (p = 0.00098, Figure 3E). To look for regulatory factors to the gene that we are interested in, and use the regulatory relationship to provide more evidence for the predicted survival effects, we searched for related miRNAs in the miRDB (Chen and Wang, 2020) database with PARP3 as the target gene, and performed the prognostic analysis for all these miRNAs in ToPP. Interestingly, one of the PARP3-related miRNA hsa-miR-98-3p showed that patients with higher expression of hsa-miR-98-3p indicated a poorer prognosis (p = 0.012, Figure 3F). As miRNAs mediated silencing and repression of their targeted mRNA molecules, the observations of both the higher expression of has-miR-98-3p and the lower expression of its target gene PARP3 associated with a poorer prognosis provide us higher confidence of the effects of the has-miR-98-3p-PARP3 regulatory relationship to survival. In addition, we found that the expression of hsa-miR-98-3p in tumor tissues was significantly higher than that in adjacent tissues (wilcoxon rank sum test, p < 0.001, Figure 3G) in patients with BRCA, but PARP3 showed the opposite expression pattern (wilcoxon rank sum test, p < 0.001, Figure 3H). Correlation analysis of hsa-miR-98-3p and PARP3 expression in tumor tissues of patients with BRCA also suggested the significant correlation between the expression levels of hsa-miR-98-3p and PARP3 (spearman correlation, p < 2.2e-16, R = −0.36 Figure 3I). Previous studies have found that hsa-miR-98-5p overexpression could increase breast cancer cells proliferation, migration, and invasion indicating that hsa-miR-98-5p functions as an oncogenic role in breast cancer (Wang et al., 2018). However, the role of hsa-miR-98-3p in breast cancer was not clear. Through our case study result here, we might speculate that hsa-miR-98-3p could have affected the repair of chromosomal DNA single-strand breaks by negatively regulating PARP3, thereby affecting the prognosis of patients with BRCA. Or such analysis result could justify more functional experimental validation. This case study also demonstrates that ToPP can provide multiple-omic molecular feature selection in prognostic research.

#### Automatic gene set feature optimization and validation of a prognostic model for a certain cancer type in ToPP

Owing to the complexity and heterogeneity of diseases, it remains a challenge to accurately predict the prognosis of all patients using a single marker. Therefore, it is desirable to combine different signatures to construct a versatile prognostic model to predict outcome. However, building a stable prognostic model requires sophisticated statistical skills and profound medical knowledge which may be cumbersome for researchers. Here, we illustrate a case study to build a prognostic model automatically with a set of gene signatures to predict prognosis for one cancer type using ToPP. Let’s take liver cancer as an example. ToPP provides two independent liver cancer cohorts with gene expression data and complete clinical follow-up data (OS), one cohort with 365 samples obtained from TCGA, and the other cohort with 232 samples from the LIRI-JP project downloaded from ICGC Data Portal (Fujimoto et al., 2016). We used the normalized read count values available in the gene expression file. We set the TCGA-LIHC cohort as the training set and LIRI-JP cohort as the validation set. Firstly, we selected univariate analysis function in ToPP to screen out the top 10 genes (KPNA2, G6PD, SFPQ, SOCS2, EZH2, RAMP3, UCK2, GTPBP4, CBX2, and LIMS2) which are most relevant to the prognosis (OS) of liver cancer in gene expression level (Table S3). Then, the expressions of these genes were used as candidate features for model construction. We chose AIC as model selection in a stepwise (select backward) algorithm. Eventually, a four-gene (karyopherin subunit alpha 2(KPNA2), suppressor of cytokine signaling 2(SOCS2), GTP-binding protein 4(GTPBP4), and chromobox 2(CBX2)) signature model was constructed by multivariate analysis showing that they were independent prognostic factors for liver cancer (Table 3).

**Table 3:** Univariate and multivariate analysis for a four-gene signature for the prognosis of liver cancer

symbol	Univariate		Multivariate	
	HR(%95CI)	p value	HR(%95CI)	p value
CBX2	1.90:(1.33–2.69)	2.90E-04	1.15:(1.02–1.30)	2.48E-02
GTPBP4	2.38:(1.663.41)	1.29E-06	1.49:(1.06–2.09)	2.20E-02
KPNA2	2.28:(1.60–3.27)	3.43E-06	1.31:(1.04–1.66)	2.18E-02
SOCS2	0.457:(0.319–0.654)	1.13E-05	0.82:(0.72–0.93)	1.46E-03

**Figure 4:**
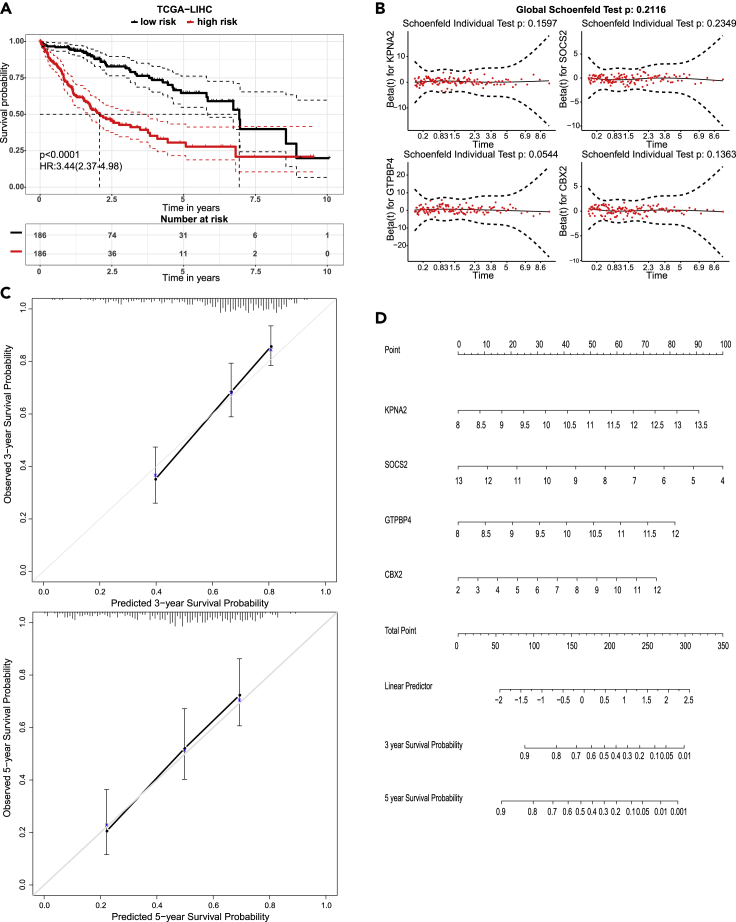
Four-gene signature-predictive prognosis model in liver cancer (A) The risk score calculated with KPNA2, SOCS2, GTPBP4, and CBX2 could assess patient prognosis (log-rank test, p < 0.0001). (B) Four covariates fit proportional hazards (PH) assumption for a Cox regression model (Schoenfeld test, p = 0.2116). (C) 3-years and 5-years calibration curves showing model-predicted survival vs. observed fraction. (D) Nomogram based on four-gene signature predicting patient outcome in 3 and 5 years.

To prove the effect of our automatically generated prognosis model, we validated the model by the following analyses. The C-index with 1000 bootstrap replications in the training cohort (TCGA-LIHC) was 0.724 and 0.752 in validation cohort (LIRI-JP). We stratified the samples into two groups (high-risk or low-risk) according to the median value of the risk score=0.272∗KPNA2−0.202∗SOCS2+0.396∗GTPBP4+0.138∗CBX2. KM curve was drawn with these two groups, and the log-rank test was with p < 0.0001 (Figure 4A). Besides, Cox model diagnosis and validation was performed simultaneously. The results show that all covariates satisfy the proportional hazards (PH) assumption and the global test of this Cox regression model is 0.212, which means the model as a whole meets the PH assumption (Figure 4B). A nomogram that incorporated all of the significant independent factors for predicting the 3-year and 5-year survival rates in the testing cohort was established (Figure 4D) and the associated calibration curves from the nomograms at 3 and 5 years are shown in Figure 4C.

#### Investigation of mutation-expression regulation by sub-stratification prognosis analysis in ToPP: patients with high expression of HIST2H3C have poor prognosis only in IDH1 mutation subgroup of lower-grade glioma

**Figure 5:**
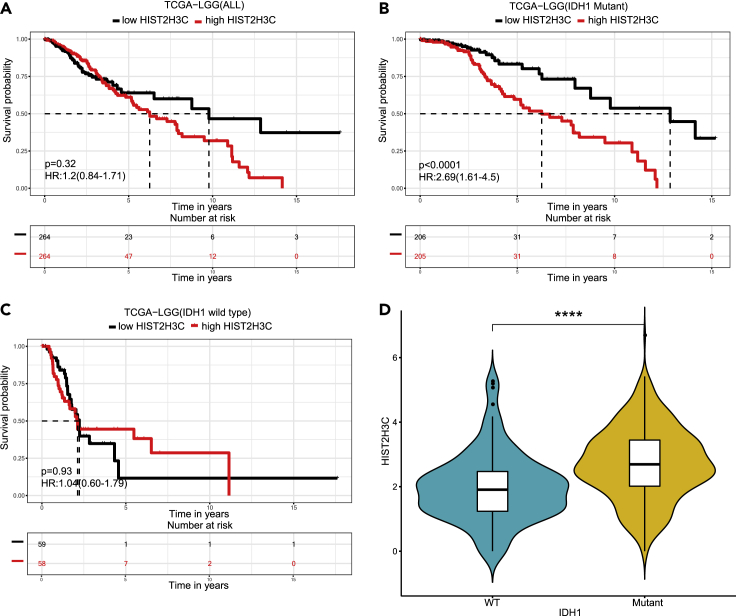
Patients with lower-grade gliomas (LGG) with high expression of HIST2H3C have poor prognosis only in IDH1 mutation subgroup (A) Prognosis analysis of HIST2H3C mRNA expression in 528 patients with LGG shows no significant difference (log-rank test, p = 0.32) for patient outcome. (B) High expression of HIST2H3C has poor prognostic (log-rank test, p < 0.0001, HR: 2.69 95%CI: 1.61–4.5) in IDH1 mutation subgroup. (C) There is no significant difference (log-rank test, p = 0.93) in IDH1 wild-type subgroup. (D) Patients with LGG have higher expression of HIST2H3C in IDH1 mutation subgroup than IDH1 wild-type subgroup (wilcoxon rank-sum rank sum test, ∗∗∗∗, p < 0.0001).

The featured combination module in ToPP can help us to have better stratification of patients and understand the prognostic effects. As an example, here we used the mutation of isocitrate dehydrogenase 1(IDH1) as a condition for patient stratification and prognosis analysis. IDH1 is one of the most commonly mutated genes in diffuse lower-grade gliomas (LGGs), and it is significantly related to the prognosis of LGG (Deng et al., 2019). IDH1/2 mutations are thought to result in hypermethylated histones and DNA (Raineri and Mellor, 2018). In order to investigate the effect of IDH1 and histone status on the prognosis of patients with LGG, here, we selected H3 clustered histone 14 (H3C14, also known as HIST2H3C), which plays a central role in transcription regulation, DNA repair, DNA replication, and chromosomal stability as an example (Herranz et al., 2016). Firstly, we retrieved the effect of HIST2H3C expression level on prognosis in LGG, and found that there was no significant (p = 0.32, Figure 5A) difference between high expression and low expression groups in overall survival (OS). In order to examine whether HIST2H3C expression has an effect on prognosis when IDH1 is mutated, we divided the patients into two groups: IDH1 mutation group and IDH1 wild-type group in ToPP. The results showed that patients with higher expression of HIST2H3C have poorer prognosis in overall survival (OS), progression-free interval (PFI), disease-specific survival (DSS), disease-free interval (DFI), and relapse-free survival (RFS) in the IDH1-mutated group (p < 0.0001, HR = 2.69; p = 0.00012, HR = 2.0; p < 0.0001, HR = 3.23; p = 0.017, HR = 3.26; p = 0.016, HR = 1.64, Figures 5B and S1). However, this phenomenon was not found in IDH1 wild-type group (p = 0.93, Figure 5C). Further investigation found that the isocitrate dehydrogenase (IDH) mutant is related to increased methylation of histone lysine residues (Venneti et al., 2013), while, methylation of some lysine and arginine residues of histones results in transcriptional activation (Whetstine, 2010). Moreover, we found that the expression of HIST2H3C in patients with LGG with IDH1 mutation was significantly higher than that in IDH1 wild-type patients (wilcoxon rank sum test, p < 0.001, Figure 5D). The above evidence shows that HIST2H3C affects the prognosis of patients through a joint effect with IDH1 mutation. In subsequent mechanism or drug research, it may suggest a new approach to treat LGG by inhibiting HIST2H3C expression when the IDH1 gene is mutated.

## Discussion

ToPP is an interactive web application for survival analysis for multi-omics data (genome, transcriptome, proteome, epigenome, and clinical data) of 68 cancer projects from the TCGA, CPTAC, and ICGC Data Portal. It collects comprehensive, standardized data. Therefore, it is at the first place in an affluent data repository for versatile cancer prognostic features. Genes or proteins can be searched in ToPP to check on its prognostic value in multiple published datasets.

ToPP provides flexible and comprehensive prognosis analysis modules, such as univariate, multivariate, combined, patient subgroup stratification survival analysis, self-designed prognostic model construction, and pan-cancer prognosis analysis module. Also, users can upload their own data for prognostic modeling and analysis.

All the data resources and analysis functions are provided in a friendly and simple interface for the convenient application to experimental cancer researchers. For more advanced computational biologists, the scripts in our GitHub account with full resources are also provided.

### Limitations of the study

There are also some limitations in ToPP. It only contains relatively common types of tumors, which may not be suitable for some specific types of tumors or other types of diseases. The clinical subtype annotation information of some tumors is not comprehensive enough, such as liver cancer or colon adenocarcinoma and so on. The molecular subgroup does not include the mutation sites, alternative splicing, or integrated signatures such as tumor mutation burden, which we notice are being increasingly studied in cancer prognosis. The analytical conclusions obtained by our platform are all data-driven which may lack more biological explanations and experiments to validate the results. We will keep expanding the data repository and function panels of ToPP to make it a sustainable resource for cancer researchers.

## STAR★Methods

### Key resources table

**Table:** 

REAGENT or RESOURCE	SOURCE	IDENTIFIER
**Deposited data**		

Code for analyses	This paper	https://github.com/kbvstmd/ToPP
multi-omics data and clinical data	This paper	http://www.biostatistics.online/topp/index.php

**Software and algorithms**		

R 3.5.0	R Core Team	https://www.rproject.org/
MySQL 5.7.17	Oracle Corporation	https://www.mysql.com/
PHP 7.0.12	The PHP Group	https://www.php.net/
rms 5.4.1	(Núñez et al., 2011)	https://cran.r-project.org/web/packages/rms/index.html
survminer 0.4.6	Alboukadel Kassambara (alboukadel.kassambara@gmail.com)	https://cran.r-project.org/web/packages/survminer/index.html
stringr 1.4.0	(Wickham et al., 2019)	https://cran.r-project.org/web/packages/stringr/index.html
data.Table 1.12.8	Matt Dowle (mattjdowle@gmail.com)	https://cran.r-project.org/web/packages/data.table/index.html
dplyr 0.8.5	(Wickham et al., 2019)	https://cran.r-project.org/web/packages/dplyr/index.html
forestplot 1.9	Max Gordon (max@gforge.se)	https://cran.r-project.org/web/packages/forestplot/index.html
ggpubr 0.2.5	Alboukadel Kassambara (alboukadel.kassambara@gmail.com)	https://cran.r-project.org/web/packages/ggpubr/index.html
ggsignif 0.6.0	Constantin Ahlmann-Eltze (artjom31415@googlemail.com)	https://cran.r-project.org/web/packages/ggsignif/index.html

### Resource availability

#### Lead contact

Further information and requests for resources and reagents should be directed to and will be fulfilled by the lead contact, Lu Xie (luxiex2017@outlook.com).

#### Materials availability statements

This study did not generate new unique reagents.

#### Data and code availability

• All the public data including multi-omics data and clinical data for all the samples can be obtained from the ToPP web server (http://www.biostatistics.online/topp/index.php).
• All original code has been deposited at https://github.com/kbvstmd/ToPP and is publicly available as of the date of publication.
• Any additional information required to reanalyze the data reported in this paper is available from the lead contact upon request.

### Method details

#### Data resource

Multi-omic (Genomic, Transcriptomic, Proteomic, Epigenomic) and clinical data for multiple tumor types from TCGA, ICGA and CPTAC projects were integrated in the ToPP platform (Tables 2 and S1). The genomic data mainly included three types: somatic mutation, Copy Number Variation (CNV) and gene fusion. Somatic mutation data were downloaded from UCSC Xena(Sanborn et al., 2011) and ICGC data portal. CNV data were extracted from cBioPortal data portal(Cerami et al., 2012) using the R-package ‘cgdsr’ (https://github.com/cBioPortal/cgdsr) and ICGC data portal while gene fusion datasets were obtained from TumorFusions(Xin et al., 2018). The Transcriptomic data included mRNA expression, miRNA expression and lncRNA expression. The mRNA expression and miRNA expression data were downloaded from UCSC Xena and ICGC data portal, and the LncRNA expression data were collected from TANRIC(Li et al., 2015). Proteomic data included reverse phase protein array (RPPA) data and MS-based global proteomic data. The RPPA data were acquired from UCSC Xena and MS proteomic data(isobaric Tags for Relative and Absolute Quantification, iTRAQ) in Breast invasive carcinoma (BRCA) (Mertins et al., 2016), Colon adenocarcinoma (COAD) (B. Zhang et al., 2014), Ovarian serous cystadenocarcinoma (OV) (Zhang et al., 2016a) and Rectum adenocarcinoma (READ) (B. Zhang et al., 2014) were extracted from CPTAC using the R-package ‘TCGA-Assembler-2’(Wei et al., 2018). Epigenome data (DNA methylation data) were downloaded from UCSC Xena and ICGC data portal. Clinical data were collected from UCSC Xena, cBioPortal data portal and ICGC data portal, which included overall survival(OS), progression-free interval(PFI), disease-specific survival(DSS), disease-free interval(DFI), Relapse Free Survival(RFS) (Liu et al., 2018) vital status, tumor stage, age, height, weight, gender, race, lymphatic invasion status, lymph node status, primary tumor pathologic spread, molecular subtypes and immune subtypes (Hoadley et al., 2018) (Table 1). Pan-cancer data were downloaded from PanCanAtlas(Weinstein et al., 2013) in UCSC Xena.

#### Data preprocessing

The CNV data acquired from cBioPortal data portal were normalized to −2, −1, 0, 1, 2. Here we defined - 2 as CNV loss and 2 for CNV gain. In the meantime, the CNV data were downloaded from ICGC data portal which contains the type information of CNV (gain, loss, etc). Here we only kept the information of CNV for type gain and loss. Somatic mutation data were normalized into 1 and 0, where 1 represents the gene with at least one site mutated, and 0 represents no mutation in this gene. Similarly, gene fusion data was normalized to 1 or 0. Here, 1 indicates the observation of a fusion event in this gene and 0 is the opposite. The methylation value of a gene was determined by the mean beta value of all CpG sites in this gene, and we defined beta values greater than 0.8 as hypermethylation and lower than 0.2 as hypomethylation by default. Users can also customize the thresholds of beta values by selecting hypermethylation or hypomethylation sites in survival analysis. For all of the above data types, we only kept tumor samples. For other data types, such as expression data, we kept both tumor and normal samples.

#### Survival analysis

##### Subgroup selection

With the advance of precision medicine, developing drugs that target a subset of patients with particular features contributing to poor survival can be beneficial to these patients. The function of “subgroup selection” in ToPP is aimed to meet this demand. It mainly includes molecular subgrouping and clinical subgrouping. Molecular subgrouping function allows users to classify mutation subgroups, CNV subgroups and methylation subgroups according to one or more genes to the user’s interest. Clinical subgrouping includes not only the traditional classification such as gender, race or tumor stage, but also the molecular label subgroups, which have been widely applied in clinic, such as the HER2 positive or negative subgroup in breast cancer. Also, the distribution of the clinical features for each molecular stratified group will be show to help identify any potential bias due to some clinical confounding variables.

#### Univariate analysis

Univariate analysis was performed with log rank test. For continuous variables such as gene or protein expression, patients can be divided into two groups according to the median value of the variable, quantile or the ‘best-cut’ value. Here, we calculated the log rank p values for the filtering of each sample and kept at least 10% of samples for each group, and the cut point with the lowest p value was represented as the “best-cut”. For categorical variables such as somatic mutation, CNV or gene fusion, patients were divided into two groups by the binarized classification. Kaplan-Meier (KM) survival curves(Kleinbaum and Klein, 2012) were then drawn by the divided groups, the hazard ratio and the 95% confidence interval information were also included in the survival plot.

#### Combination analysis

Combination analysis is to analyze the prognosis effects by considering two features. These two features can be in the same level of data such as the expression of two genes, or they can be in different levels. For instance, if a researcher wants to know the prognostic impact of gene A expression in combination with gene B mutant. Then, all the patients were divided into four groups (high expression + mutation group, high expression + wild type group, low expression + mutation group and low expression + wild type group) according to the threshold of two features, and then log-rank test was performed to test whether there was significant difference between any of two groups, separately. Co-inactivation of one gene pair which results in better survival may provide evidence for synthetic lethality interaction prediction(Lee et al., 2018).

#### Multivariate analysis and prognostic modeling

Cox regression (or Cox proportional hazards model) is used for multivariate analysis(Christensen, 1987). When constructing a prognostic model, we firstly fit a naive Cox model including all covariates. Then, redundant or irrelevant variables from the model should be removed. Interaction between covariates and time-dependent covariates should also be considered in the modeling process (Austin et al., 2020). Therefore, stepwise regression is performed to select the variables of the model and is evaluated by the Akaike information criterion (AIC) value. For diagnostic model, we mainly included the following three aspects: 1) testing the proportional hazards (PH) assumption, 2) examining influential observations (or outliers), 3) detecting nonlinearity in relationship between the log hazard and the covariates. In order to test the model assumptions, residuals method was used. The common residuals for the Cox model included: 1) Schoenfeld residuals to test the proportional hazards assumption; 2) Martingale residual to assess nonlinearity; 3) Deviance residual (symmetric transformation of the martinguale residuals), to examine influential observations(Xue and Schifano, 2017). Covariates conversion such as nonlinear transformations should be performed if it is necessary. Finally, we used Concordance index (C-index) (Harrell et al., 1996) to evaluate the effect of the model.

#### Pan-cancer analysis

In order to reduce the impact of tissue specificity on the data, we used a specially standardized dataset named the Pan-Cancer Atlas(Hoadley et al., 2018), which has normalized and removed batch effects of the data, making the data comparable. In addition, since the expression of genes in various types of tumors may be quite different, when doing the pan-cancer prognostic analysis, we first divided the patients into high gene expression group and low gene expression group according to the median gene expression in each type of tumor, separately. Then, all the high expression samples were combined as the pan-cancer high group, so is the low expression samples. After that the log rank test was performed between these two groups.

### Quantification and statistical analysis

The ToPP web interface was developed using HTML5 and PHP script (version 7.0.12) in the Bootstrap framework. JavaScript and jQuery were also used to perform dynamic web services with js library such as DataTables.js and select2.js. The database was implemented in MySQL (version 5.7.17) and deployed in Apache web server running on the CentOS 6.5 system. Data preprocessing and data analyses were performed by the R scripts (version 3.5.0) with several R packages (rms v5.4.1, survminer v0.4.6, stringr v1.4.0, data.table v1.12.8, dplyr v0.8.5, forestplot v1.9, ggpubr v0.2.5, ggsignif v0.6.0). Wilcoxon rank sum test was used to compare two groups, and p < 0.05 was considered as significant difference. Significance levels p < 0.05, p < 0.01 and p < 0.001 are noted using asterisks ∗, ∗∗, and ∗∗∗, respectively.
