# Supplementary material for: ToPP: Tumor online prognostic analysis platform for prognostic feature selection and clinical patient subgroup selection
Source: iScience. 2022 Apr 4;25(5):104190. doi: 10.1016/j.isci.2022.104190 (PMC9035726; doi:10.1016/j.isci.2022.104190)
Supplement: Document S1. Figure S1 and Tables S1–S3 [file mmc1.pdf]

iScience, Volume *25*

Supplemental information

ToPP: Tumor online prognostic analysis platform for prognostic feature selection and clinical patient subgroup selection

Jian Ouyang, Guangrong Qin, Zhenhao Liu, Xingxing Jian, Tieliu Shi, and Lu Xie

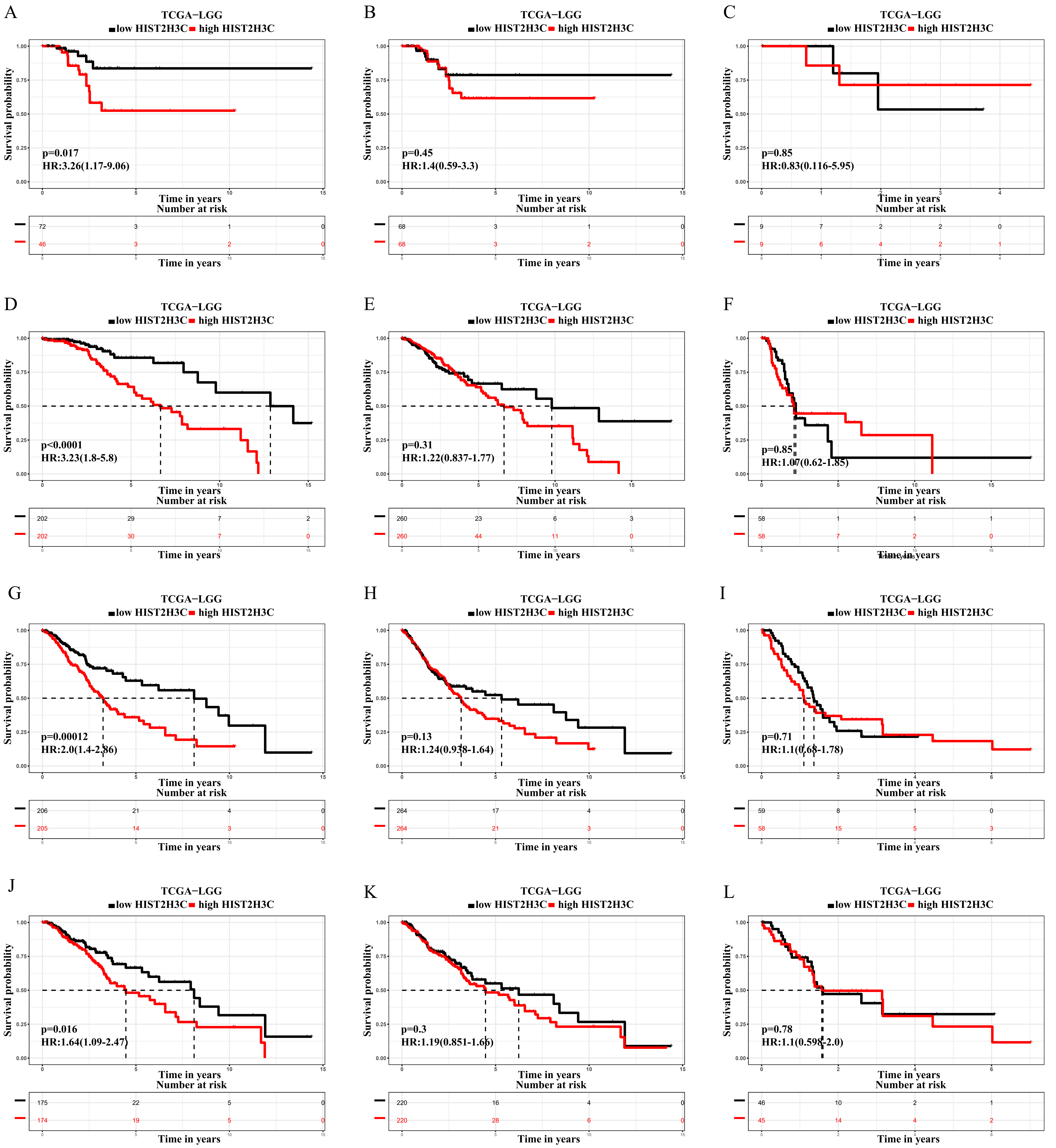
A
TCGA−LGG
low HIST2H3C high HIST2H3C
Survival probability
p=0.017
HR:3.26(1.17-9.06)
Time in years
Number at risk
72 3 1 0
46 3 2 0
Time in years
B
TCGA−LGG
low HIST2H3C high HIST2H3C
Survival probability
p=0.45
HR:1.4(0.59-3.3)
Time in years
Number at risk
68 3 1 0
68 3 2 0
Time in years
C
TCGA−LGG
low HIST2H3C high HIST2H3C
Survival probability
p=0.85
HR:0.83(0.116-5.95)
Time in years
Number at risk
9 7 2 2 0
9 6 4 2 1
Time in years
D
TCGA−LGG
low HIST2H3C high HIST2H3C
Survival probability
p<0.0001
HR:3.23(1.8-5.8)
Time in years
Number at risk
202 29 7 2
202 30 7 0
Time in years
E
TCGA−LGG
low HIST2H3C high HIST2H3C
Survival probability
p=0.31
HR:1.22(0.837-1.77)
Time in years
Number at risk
260 23 6 3
260 44 11 0
Time in years
F
TCGA−LGG
low HIST2H3C high HIST2H3C
Survival probability
p=0.85
HR:1.07(0.62-1.85)
Time in years
Number at risk
58 1 1 1
58 7 2 0
Time in years
G
TCGA−LGG
low HIST2H3C high HIST2H3C
Survival probability
p=0.00012
HR:2.0(1.4-2.86)
Time in years
Number at risk
206 21 4 0
205 14 3 0
Time in years
H
TCGA−LGG
low HIST2H3C high HIST2H3C
Survival probability
p=0.13
HR:1.24(0.938-1.64)
Time in years
Number at risk
264 17 4 0
264 21 3 0
Time in years
I
TCGA−LGG
low HIST2H3C high HIST2H3C
Survival probability
p=0.71
HR:1.1(0.68-1.78)
Time in years
Number at risk
59 8 1 0
58 15 5 3
Time in years
J
TCGA−LGG
low HIST2H3C high HIST2H3C
Survival probability
p=0.016
HR:1.64(1.09-2.47)
Time in years
Number at risk
175 22 5 0
174 19 5 0
Time in years
K
TCGA−LGG
low HIST2H3C high HIST2H3C
Survival probability
p=0.3
HR:1.19(0.851-1.66)
Time in years
Number at risk
220 16 4 0
220 28 6 0
Time in years
L
TCGA−LGG
low HIST2H3C high HIST2H3C
Survival probability
p=0.78
HR:1.1(0.598-2.0)
Time in years
Number at risk
46 10 2 1
45 14 4 2
Time in years

Figure S1. The effect of HIST2H3C expression on prognosis for disease-free interval (DFI), disease-specific survival (DSS), progression-free interval (PFI) and relapse free survival (RFS) in the IDH1 mutated subgroup, IDH1 wild-type subgroup and all LGG patients, separately, Related to Figure 5.

Table S1. Multi-omics data of tumors from ICGC included in ToPP, Related to Table 1.

ID	project	Genome		Transcriptome	Epigenome	Clinical data
		Mutation	CNV	mRNA	Methylation	Phenotype &Follow up
BOCA-FR	Soft Tissue cancer	√		√		√
BOCA-UK	Bone Cancer	√		√		√
BRCA-FR	Breast Cancer	√	√			√
BRCA-KR	Breast Cancer	√	√			√
BTCA-JP	Biliary Tract Cancer	√				√
BTCA-SG	Biliary Tract Cancer	√				√
CLLE-ES	Chronic Lymphocytic Leukemia	√	√	√	√	√
CMDI-UK	Chronic Myeloid Disorders	√				√
COCA-CN	Colorectal Cancer	√		√		√
EOPC-DE	Early Onset Prostate Cancer	√		√		√
ESAD-UK	Esophageal Adenocarcinoma	√		√		√
ESCA-CN	Esophageal Cancer	√				√
GACA-CN	Gastric Cancer	√				√
LAML-KR	Acute Myeloid Leukemia	√				√
LICA-FR	Liver Cancer	√		√		√
LINC-JP	Liver Cancer	√				√
LIRI-JP	Liver Cancer	√	√			√
LMS-FR	Soft tissue cancer - Leiomyosarcom	√				√
LUSC-KR	Lung Cancer	√	√			√
MALY-DE	Malignant Lymphoma	√				√
MELA-AU	Skin Cancer	√				√
NKTL-SG	Blood Cancer - T-cell and NK-cell lymphoma	√				√
ORCA-IN	Oral Cancer	√				√
OV-AU	Ovarian Cancer	√	√	√	√	√
PACA-AU	Pancreatic Cancer	√	√	√	√	√
PACA-CA	Pancreatic Cancer	√	√	√		√
PAEN-AU	Pancreatic Cancer Endocrine neoplasms	√	√	√	√	√
PAEN-IT	Pancreatic Endocrine Neoplasms	√		√		√
PBCA-DE	Pediatric Brain Cancer	√			√	√
PRAD-FR	Prostate Cancer	√	√			√
PRAD-UK	Prostate Adenocarcinoma	√		√		√
RECA-EU	Renal Cell Cancer	√	√			√
SKCA-BR	Skin Adenocarcinoma	√		√		√
THCA-SA	Thyroid Cancer	√				√
UTCA-FR	Uterine Cancer - Carcinosarcoma	√				√

Table S2. Subset for TCGA projects classified by the Pan-Cancer Atlas, Related to Figure 2.

subset	TCGA projects
thoracic	lung adenocarcinoma(LUAD)
	lung squamous cell carcinoma(LUSC)
	mesothelioma(MESO)
endocrine	thyroid carcinoma(THCA)
	adrenocortical carcinoma(ACC)
head and neck	head and neck squamous cell carcinoma(HNSC)
eye	uveal melanoma(UVM)
cancers of the central nervous system	glioblastoma multiforme(GBM)
	brain lower-grade glioma(LGG)
hematologic and lymphatic malignancies	acutemyeloid leukemia(LAML)
	lymphoid neoplasm diffuse large B celllymphoma(DLBC)
	andthymoma(THYM)
soft Tissue	sarcoma(SARC)
	uterine carcinosarcoma(UCS)
skin	skin cutaneous melanoma(SKCM)
gynecologic	ovarian(OV)
	uterine corpus endometrial carcinoma(UCEC)
	cervical squamous cell carcinoma and endo-cervical adenocarcinoma(CESC)
	breast invasive carcinoma(BRCA)
urologic	bladder urothelial carcinoma(BLCA)
	prostate adenocarcinoma(PRAD)
	testicular germ cell tumors(TGCT)
	kidney renal clear cell carcinoma(KIRC)
	kidney chromophobe(KICH)
	kidney renal papillary cell carcinoma(KIRP)
developmental gastrointestinal	liver hepatocellular carcinoma(LIHC)
	pancreatic adenocarcinoma(PAAD)
	cholangiocarcinoma(CHOL)
core gastrointestinal	esophageal carcinoma(ESCA)
	stomach adenocarcinoma (STAD)
	colon adenocarcinoma(COAD)
	rectum adenocarcinoma(READ)

Table S3. Top 10 gene which are most relevant to the prognosis (OS) of LIHC in gene expression level, Related to Figure 4 and Table 3.

gene symbol	Hazard Ratio (%95CI)	log-rank P-value
GTPBP4	2.38:(1.66-3.41)	0.00000129
SFPQ	2.28:(1.6-3.26)	0.00000283
KPNA2	2.28:(1.6-3.27)	0.00000343
SOCS2	0.457:(0.319-0.654)	0.0000113
RAMP3	0.464:(0.325-0.662)	0.000015
EZH2	2.15:(1.5-3.07)	0.0000184
UCK2	2.01:(1.41-2.86)	0.0000853
CBX2	1.9:(1.33-2.69)	0.000286
G6PD	1.86:(1.31-2.64)	0.000419
LIMS2	0.54:(0.38-0.766)	0.00046
